# Perovskite Solar Cells Fabricated by Using an Environmental Friendly Aprotic Polar Additive of 1,3-Dimethyl-2-imidazolidinone

**DOI:** 10.1186/s11671-017-2391-3

**Published:** 2017-12-19

**Authors:** Lili Zhi, Yanqing Li, Xiaobing Cao, Yahui Li, Xian Cui, Lijie Ci, Jinquan Wei

**Affiliations:** 1 School of Materials Science and Engineering, Shandong University, Jinan, 250061 Shandong People’s Republic of China; 2 Department of Physics, Changji College, Changji, 831100 Xinjiang People’s Republic of China; 3 Key Lab for Advanced Materials Processing Technology of Education Ministry, Tsinghua University, Beijing, 100084 People’s Republic of China; 4 State Key Lab of New Ceramic and Fine Processing, Tsinghua University, Beijing, 100084 People’s Republic of China; 5 School of Materials Science and Engineering, Tsinghua University, Beijing, 100084 People’s Republic of China

**Keywords:** Perovskite solar cell, Aprotic polar solvent, Lewis acid–base adduct, 1,3-Dimethyl-2-imidazolidinone

## Abstract

Perovskite solar cells (PSCs) have great potentials in photovoltaics due to their high power conversion efficiency and low processing cost. PSCs are usually fabricated from PbI_2_/dimethylformamide solution with some toxic additives, such as *N*-methyl pyrrolidone and hexamethylphosphoramide. Here, we use an environmental friendly aprotic polar solvent, 1,3-dimethyl-2-imidazolidinone (DMI), to fabricate perovskite films. By adding 10 vol% DMI in the precursor solution, high-quality perovskite films with smooth surface are obtained. By increasing annealing temperature from 100 to 130 °C, the average grain size of the perovskite increases from ~ 216 to 375 nm. As a result, the efficiency of the PSCs increases from 10.72 to 14.54%.

**Electronic supplementary material:**

The online version of this article (10.1186/s11671-017-2391-3) contains supplementary material, which is available to authorized users.

## Background

Recently, organometallic halide perovskite solar cells (PSCs) have attracted great attentions due to rapid growth of power conversion efficiency (PCE) and low processing cost [1–8]. Currently, the perovskite solar cells are mainly fabricated through solution-based processing, including one-step [9–12], two-step [13, 14], and additive-assisted deposition methods [15, 16]. The two-step method has been widely used for achieving high-efficient perovskite solar cells. In the traditional two-step method, the CH_3_NH_3_PbI_3_ perovskite (MAPbI_3_) is formed through intercalation of CH_3_NH_3_I (MAI) into the PbI_2_ lattice, which usually leads to rough surface due to volume expansion and existence of some small grains on the perovskite films [17, 18].

Generally, dimethylformamide (DMF) is used as solvent for preparing PbI_2_ and MAPbI_3_ films. The volatile DMF solvent has a high saturated vapor pressure, which makes the PbI_2_ crystallize rapidly during spin-coating of the PbI_2_/DMF solution, so it is hard to control the crystallinity of the PbI_2_ films. The morphology of the perovskite film depends on the PbI_2_ strongly. In order to obtain smooth and dense perovskite films with large grains, researchers usually added some additives to the PbI_2_/DMF precursor solution. For example, Zhang et al. reported preparation of a smooth MAPbI_3_ film by incorporating 4-tert-butylpyridine (TBP) into the PbI_2_/DMF precursor solution [19]. Li et al. mediated the nucleation and grain growth pathway to obtain large perovskite grains in micrometer scale by introducing an acetonitrile to the PbI_2_/DMF solution [20]. Recently, Lewis acid–base adduct approach was also used to fabricate high-quality perovskite films. Some aprotic polar solvents, such as DMF, *N*,*N*-dimethyl sulfoxide (DMSO), *N*-methyl pyrrolidone (NMP), and hexamethylphosphoramide (HMPA), have been used as Lewis base solvents to improve the quality and performance of the perovskite solar cells [21–23]. Lee et al. [24] pointed out that aprotic polar solvents, containing oxygen, sulfur, or nitrogen ligands, were Lewis bases, which can form Lewis acid–base adducts of PbI_2_·xSol with PbI_2_ through dative bonds. The Lewis adducts of PbI_2_·xSol lead to high-quality perovskite films and high-efficient PSCs. However, the above aprotic polar solvents are toxic, which harm health and environments.

1,3-Dimethyl-2-imidazolidinone (DMI) is also an aprotic polar solvent with low volatility. The DMI has a five-membered ring and a carbonyl (see Additional file 1: Figure S1). Due to the isolated electron pair on the O atom of the carbonyl, DMI can also form a Lewis adduct with PbI_2_. More importantly, the potential toxicological risk of DMI is less than carcinogen HMPA and reproductive toxicity NMP. Thus, it is a good alternative solvent to the HMPA and NMP, in forming perovskite through the Lewis adduct approach because it provides a safer working environment [25]. Here, we introduce the DMI solvent into the PbI_2_/DMF precursor solution to improve quality of the perovskite films.

## Methods

### Device Fabrication

The perovskite films and solar cells were fabricated by a modified two-step method, which has been reported in details in our previous paper [22]. In brief, a compact TiO_2_ blocking layer was spin-coated a mildly acidic solution of titanium isopropoxy solution in ethanol at 2000 rpm for 30 s on FTO substrate, followed by sintering at 500 °C for 30 min. A mesoporous TiO_2_ layer was then deposited on the blocking layer by spin-coating diluted TiO_2_ paste (Dyesol-30NRT, Dyesol) in ethanol (1:6, weight ratio) at 3500 rpm for 30 s. The FTO substrate was sintered at 500 °C for 30 min. The FTO substrate was then dropped with 1 M PbI_2_/DMF solution adding with different volume fractions of DMI and then spin-coated at 3000 rpm for 30 s. The PbI_2_ precursor film was directly dipped into a solution of CH_3_NH_3_I (MAI) in 2-propanol with a concentration of 30 mg/mL for 120 s to prepare MAPbI_3_ films and then annealed at 100 °C for 30 min. A HTM layer was then deposited by spin-coating a solution prepared by dissolving 100 mg spiro-OMeTAD, 40 μL 4-tert-butylpyridine (TBP), 36 μL of a stock solution of 520 mg/mL TFSI in acetonitrile, and 60 μL of a stock solution of 300 mg/mL FK102 dopant in acetonitrile in 1 mL chlorobenzene. Finally, a 60-nm-thick Au film was thermally evaporated on the top of HTM to form a back electrode. The active area of the electrode was fixed at 0.06 cm^2^.

### Device Characterization

The Lewis adduct of PbI_2_∙DMI and perovskite films were characterized and evaluated by X-ray diffraction (XRD, Smartlab, Rigaku), field emission scanning electron microscopy (SEM, MERLIN VP Compact), Fourier transform infrared (FTIR) spectroscopy (VERTEX 70v), and thermogravimetric analysis (TGA, Q5000IR). Impedance spectra (IS) of the PSCs were measured in the dark by an electrochemical workstation (CHI660D) under a bias voltage of 0.9 V and an alternative signal of 10 mV in a range from 1 Hz to 1 MHz. Steady-state and time-resolved photoluminescence (PL) spectra were measured by an Edinburgh FLS 920 instrument (Livingston, WL, UK). The current-voltage curves were measured in air illustrated by a solar simulator (AM 1.5G, 100 mW/cm^2^, 91195, Newport) at a scan rate of 5 mV/s.

## Results and Discussion

**Fig. 1:**
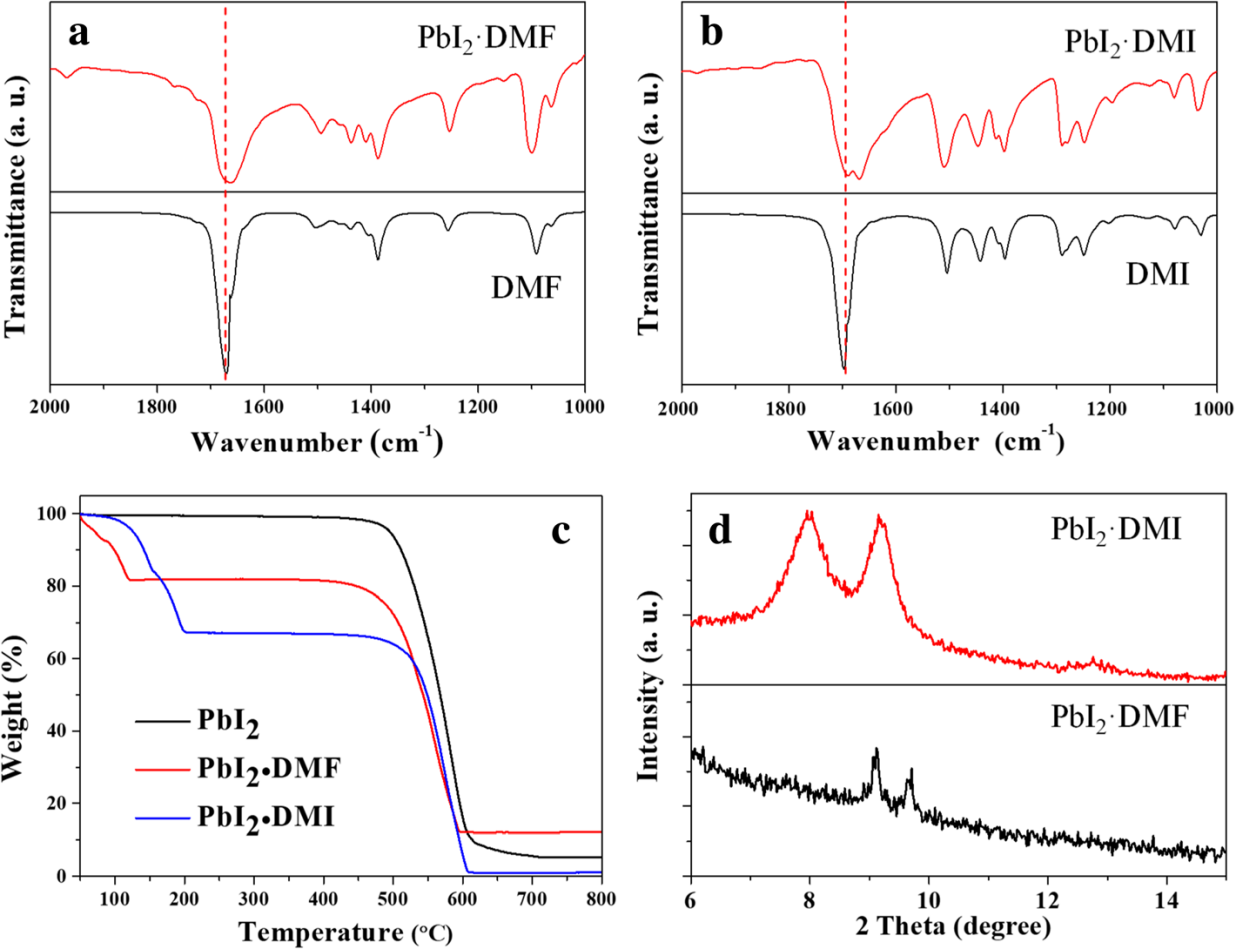
**a** Fourier transform infrared transmittance spectra of DMF and PbI_2_∙DMF. **b** Fourier transform infrared transmittance spectra of DMI and PbI_2_∙DMI. **c** Thermal gravimetric analysis of the PbI_2_ Lewis adducts. **d** XRD curves of the Lewis adducts of PbI_2_∙DMI and PbI_2_∙DMF

Figure 1a, b shows FTIR transmittance spectra of the pure DMF and DMI solvents and their corresponding Lewis adducts. The stretching vibration of C=O bonds is located at 1670 and 1697 cm^−1^ for the DMF and DMI solvents, respectively. When forming Lewis adducts, the C=O peaks separately downshift to 1658 and 1668 cm^−1^. It indicates that both of the DMI and DMF can interact with PbI_2_ through dative Pb–O bonds, which separately form Lewis adducts of PbI_2_∙DMI and PbI_2_∙DMF [26, 27]. Figure 1c shows TGA curves of PbI_2_ powder and its Lewis adducts of PbI_2_∙DMI and PbI_2_∙DMF. The PbI_2_·DMF decomposes completely to PbI_2_ at 120 °C, while PbI_2_·DMI decomposes completely at 200 °C. It indicates that the PbI_2_·DMI adduct is more stable than the PbI_2_·DMF due to the stronger molecular interaction between DMI and PbI_2_. Therefore, it inclines to form PbI_2_∙DMI when DMI exist in the PbI_2_/DMF precursor solution. Figure 1d shows XRD curves of the Lewis adducts of PbI_2_∙DMI and PbI_2_∙DMF, which are prepared from PbI_2_/DMF solution adding with and without 10 vol% DMI. The PbI_2_·DMI has two characteristic diffraction peaks at 7.97° and 9.21°, which are smaller than those of the PbI_2_∙DMF (9.12° and 9.72°).

When immersed in MAI/2-propanol solution, the Lewis adducts of PbI_2_∙DMI convert to perovskite through molecular exchange between DMI and MAI basing on following formula:

1 $$ {\mathrm{PbI}}_2\cdot \mathrm{DMI}\kern0.5em +\kern0.5em \mathrm{MAI}\kern0.5em \to \kern0.5em {\mathrm{MAPbI}}_3\kern0.5em +\kern0.5em \mathrm{DMI} $$

Additional file 1: Figure S2 shows XRD curves of the annealed perovskite films prepared by immersing Lewis adducts of PbI_2_∙DMI in the MAI/2-propanol solution for different times. The XRD peaks at 12.7° and 14.2° are assigned to (001) of the PbI_2_ and (110) of the perovskite, respectively [11, 28]. It shows that the PbI_2_∙DMI converts to perovskite completely within 2 min. There are some residual PbI_2_ in the perovskite films when the reaction time is less than 120 s.

**Fig. 2:**
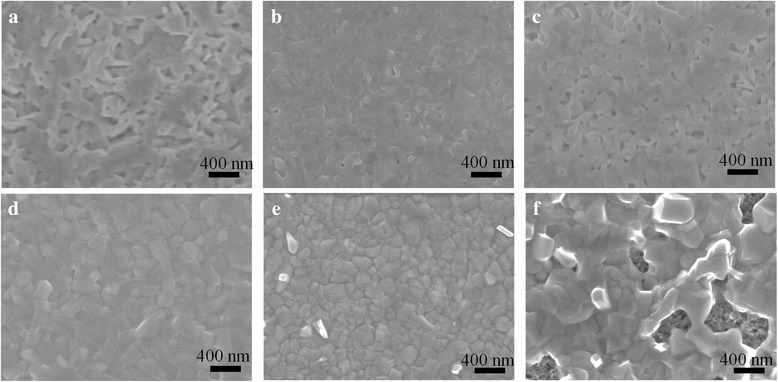
SEM images of the PbI_2_ films (top) and perovskite films (bottom) **a**, **d** without DMI, **b**, **e** 10% DMI, and **c**, **f** 20% DMI

Figure 2 shows SEM images of the PbI_2_ films and corresponding perovskite films prepared from the PbI_2_/DMF solution adding with different amounts of DMI. All the samples are annealed at 100 °C for 30 min before SEM characterization. Compared to DMF, DMI has a higher boiling point and stronger interaction with PbI_2_. Therefore, the morphology of the PbI_2_ films change evidently with the concentration of DMI. The PbI_2_ grains change from ramiform to plate-like when 10 vol% DMI is added to the PbI_2_/DMF precursor solution (see Fig. 2a, b). However, the PbI_2_ films change to porous and even discontinuous, when the DMI concentration increases to 20 vol% (Fig. 2c). The resulting MAPbI_3_ films are affected by the PbI_2_ films significantly. Thus, the perovskite film has a uniform grain and smooth surface for the sample prepared from the solution adding with 10 vol% DMI (see Fig. 2e), which is better than that without DMI. However, excessive DMI might lead to discontinuous films (see Fig. 2f), which are disadvantageous for the photovoltaic performance of PSCs.

**Fig. 3:**
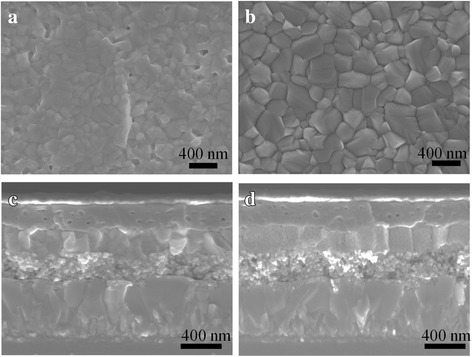
SEM images of the MAPbI_3_ films (top) and corresponding perovskite solar cells (bottom). **a**, **c** The perovskite films are prepared from PbI_2_/DMF precursor solution adding with 10 vol% DMI and annealing at 100 °C and **b**, **d** at 130 °C

In spite of uniform grain and smooth surface, the grain size of MAPbI_3_ fabricated from PbI_2_/DMF solution with 10% DMI and annealed at 100 °C is not large enough. According to the TGA curves in Fig. 1c, the DMI escapes from the Lewis adducts at higher temperature than the DMF. Herein, we increase the annealing temperature. Figure 3a, b shows top-viewed SEM images of the perovskite films prepared by annealing at 100 and 130 °C from the solution adding with 10 vol% DMI for10 min. It is clear that the grain size increases as the annealing temperature rises. The average grain sizes are 216 and 375 nm for the samples prepared from annealing temperature of 100 and 130 °C, respectively (see Additional file 1: Figure S3). Figure 3c, d shows cross-sectional SEM images of the perovskite solar cells. It shows that the perovskite solar cells have about 250-nm-thick perovskite layers. It contains only one grain in most area for the samples annealed at high temperature (130 °C), which ascribes to the larger grain size than the film thickness. When increasing the annealing temperature to 160 °C, there are some residual PbI_2_ in the perovskite films (see Additional file 1: Figure S4), which results in a poor photovoltaic performance (see Additional file 1: Figure S5 the best PCE = 8.53%).

**Fig. 4:**
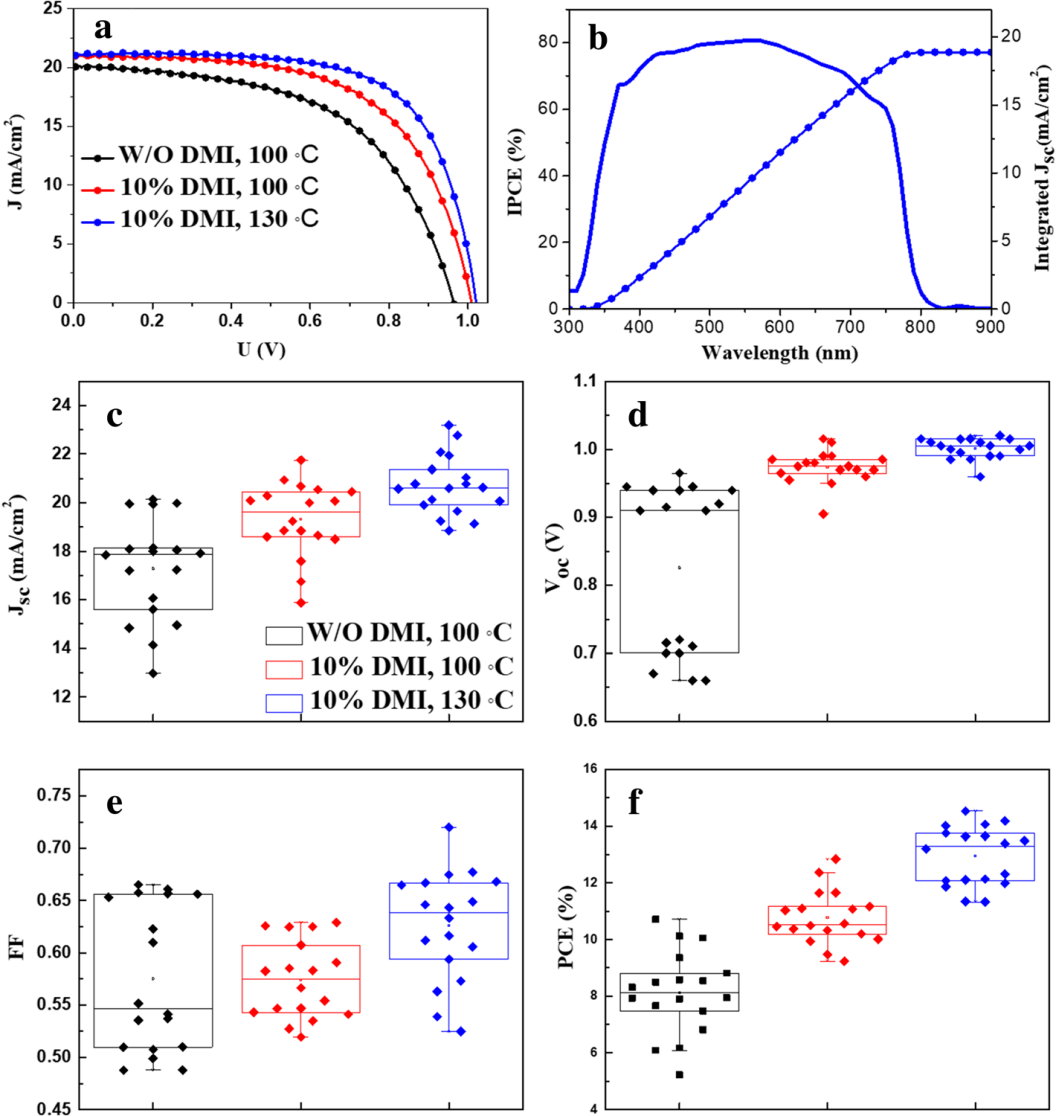
**a** *J*–*V* curves of the best PSCs fabricated from different conditions. **b** IPCE spectrum of the best PSCs fabricated from precursor solution adding with 10% DMI and annealing at 130 °C. Box charts of photovoltaic parameters obtained from light *J*–*V* curves of a series of PSCs. **c** *J*_sc_, **d** *V*_oc_, **e** FF, **f** PCE

Figure 4a shows *J*–*V* curves of the best cells fabricated from the solution adding with different DMI additives. The corresponding photovoltaic parameters are listed in Table 1. The PSCs exhibit the best photovoltaic performance with a PCE of 14.54%, a short current density (*J*_sc_) of 21.05 mA/cm^2^, an open voltage (*V*_oc_) of 1.02 V, and a fill factor (FF) of 67.72% for the samples fabricated from DMF solution adding with 10 vol% DMI and annealing at 130 °C. For the PSCs fabricated from the same precursor solution and annealing at 100 °C, the best PCE is only 12.84%. The PSCs fabricated from the solution with DMI additive have better photovoltaic performances than those from the solution without DMI (the best PCE = 10.72%, *J*_sc_ = 20.14 mA/cm^2^, *V*_oc_ = 0.97 V, FF = 55.14%). A series of photovoltaic parameters for the PSCs fabricated from different conditions exhibit the similar tendency to the best PSCs as shown in Fig. 4c–f. The devices fabricated from the solution with 10 vol% DMI and annealing at 130 °C exhibit higher PCE than that from the pure DMF. Figure 4b shows an incident photon-to-current efficiency (IPCE) result of a PSC fabricated from DMF solution adding with 10 vol% DMI, which exhibits a good quantum yield. It is noted that the integrated *J*_sc_ is about 10% lower than that obtained from the reverse scan. This discrepancy might derive from the spectral mismatch between the IPCE light source and solar simulator and from the decay of the devices during the measurement in air [29]. To check the stabilization or saturation point of photocurrent for the PSCs fabricated from the solution with 10 vol% DMI and annealing at 130 °C, we measured the steady-state current of a typical PSC at a bias voltage close to the maximum power point (0.78 V), as shown in Additional file 1: Figure S6a. The PSC shows a stable output. The device shows an evident hysteresis phenomenon in Additional file 1: Figure S6b.

**Table 1:** Photovoltaic parameters of the best PSCs fabricated from three different conditions

Sample	*J*_sc_ (mA/cm^2^)	*V*_oc_ (V)	FF (%)	PCE (%)	*R*_s_ (Ω)	*R*_rec_ (Ω)
W/O DMI, 100 °C	20.14	0.97	55.14	10.72	26.16	46.49
10% DMI, 100 °C	20.94	1.01	60.73	12.84	14.65	1206
10% DMI, 130 °C	21.05	1.02	67.72	14.54	14.30	2778

**Fig. 5:**
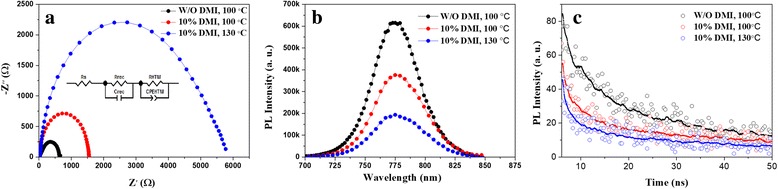
**a** Nyquist plots of the perovskite solar cells in the dark at a bias of 0.9 V. **b** Steady PL spectra of the perovskite films. **c** Time-resolved PL spectra basing on the bi-exponential decay function fabricated from three different conditions

Figure 5a shows impedance spectra of the PSCs measured in the dark at a forward bias of 0.9 V. The inset of Fig. 5a is an equivalent circuit composed of series resistance (*R*_s_), recombination resistance (*R*_rec_) and the transport resistance (*R*_HTM_) [30]. The *R*_s_ of the PSCs reduces from 26.16 to 14.30 Ω by adding 10% DMI in DMF and annealing at 130 °C compared to without DMI. The small *R*_s_ facilitates carrier transport, leading to a high *J*_sc_ [31]. On the contrary, the *R*_rec_ increases from 46.49 to 2778 Ω by adding 10 vol% DMI in DMF and annealing at 130 °C compared to pure DMF. The high *R*_rec_ effectively suppresses the charge recombination for improved device performance.

Figure 5b shows steady-state PL spectra of the MAPbI_3_ films deposited on mesoporous TiO_2_ substrate. The PL spectra are quenched for the perovskite films fabricated from the solution with 10% DMI and annealing at 130 °C, which indicates that the charges transfer effectively from MAPbI_3_ into a TiO_2_ film before they are recombined at the interface for the sample. Compared with those fabricated from the pure DMF, adding some DMI additive can improve the charge transfer. To gain more insight into charge transfer, time-resolved PL of the MAPbI_3_ films deposited on the mesoporous TiO_2_ substrate are also carried out (see Fig. 5c). The spectra are well fitted with a bi-exponential decay function:

2 $$ I(t)={A_1}^{\frac{-t}{\tau_1}}+{A_2}^{\frac{-t}{\tau_2}} $$

where *τ*_1_and *τ*_2_ are the decay time of the two decay processes, respectively. It indicates that there is a fast (*τ*_1_) and a slow (*τ*_2_) decay in the PSCs. The fast decay process is regarded as a quench effect of free carriers in the perovskite film to the electron transport layer (ETL) or HTM, whereas the slow decay process is regarded as the radiative decay [32, 33]. The *τ*_1_ reduces from 3.71 to 2.80 ns when adding 10% DMI and annealing at 100 °C. Furthermore, the *τ*_1_ reduces to 1.90 ns when adding 10% DMI and annealing at 130 °C, demonstrating that the electrons transfer faster from the perovskite film to the TiO_2_ ETL layer, as witnessed by stronger steady-state PL quenching. We believe that the enhanced charge transfer rate is ascribed to the increase of large grains and reduce of grain boundary in the perovskite films by adding DMI.

## Conclusions

We fabricated high-quality perovskite films with large grains by adding some environmental friendly DMI additives to the PbI_2_/DMF solution. It forms a compact Lewis adduct film of PbI_2_·DMI, which converts to perovskite films through molecular exchange between DMI and MAI. High-quality perovskite films with large grains are easily obtained by annealing at high temperature. The performances of the perovskite solar cells are thus improved significantly by adding 10 vol% DMI in the precursor solution and annealing at 130 °C.

## Additional file

Additional file 1: Figure S1. Molecular structures of DMF and DMI. **Figure S2.** XRD curves of the perovskite films from different immersing times. **Figure S3.** Statistical graphs of the perovskite grains prepared by annealing at different temperatures. (a) 100 °C, (b) 130 °C. **Figure S4.** XRD curves of the perovskite films prepared from three different annealing temperatures. **Figure S5.** *J*–*V* curve of the best PSC fabricated from DMI solution and annealing at 160 °C. **Figure S6.** (a) Steady-state current measured at the maximum power point (0.78 V), and (b) *J*–*V* curves under forward (black line) and reverse (red line) scans for a typical perovskite solar cell. (DOCX 295 kb)

## Abbreviations

DMF: Dimethylformamide
DMI: 1,3-Dimethyl-2-imidazolidinone
DMSO: *N*,*N*-Dimethyl sulfox
HMPA: Hexamethylphosphoramide
MAI: CH_3_NH_3_I
MAPbI_3_: CH_3_NH_3_PbI_3_
NMP: *N*-Methyl pyrrolidone
PSCs: Perovskite solar cells
