# Supplementary material for: Perovskite Solar Cells Fabricated by Using an Environmental Friendly Aprotic Polar Additive of 1,3-Dimethyl-2-imidazolidinone
Source: Nanoscale Res Lett. 2017 Dec 19;12:632. doi: 10.1186/s11671-017-2391-3 (PMC5736506; doi:10.1186/s11671-017-2391-3)
Supplement: Additional file 1: Figure S1. — Molecular structures of DMF and DMI. Figure S2. XRD curves of the perovskite films from different immersing times. Figure S3. Statistical graphs of the perovskite grains prepared by annealing at different temperatures. (a) 100 °C, (b) 130 °C. Figure S4. XRD curves of the perovskite films prepared from three different annealing temperatures. Figure S5. J–V curve of the best PSC fabricated from DMI solution and annealing at 160 °C. Figure S6. (a) Steady-state current measured at the maximum power point (0.78 V), and (b) J–V curves under forward (black line) and reverse (red line) scans for a typical perovskite solar cell. (DOCX 295 kb) [file 11671_2017_2391_MOESM1_ESM.docx]

**Perovskite solar cells fabricated by using an** **environmental friendly** **[aprotic](javascript:void(0);)**[**polar**](javascript:void(0);)**additive of 1,3-Dimethyl-2-imidazolidinone**

Lili Zhi ^1, 2^, Yanqing Li ^2^, Xiaobing Cao ^3^, Yahui Li ^3^, Xian Cui ^3^, Lijie Ci ^1*^, Jinquan Wei ^3*^

1. School of Materials Science and Engineering, Shandong University, Jinan 250061, Shandong, P.R. China
2. Department of Physics, Changji College, Changji 831100, Xinjiang, P.R. China
3. Key Lab for Advanced Materials Processing Technology of Education Ministry; State Key Lab of New Ceramic and Fine Processing; School of Materials Science and Engineering, Tsinghua University, Beijing 100084, P.R. China

*Corresponding author: [jqwei@tsinghua.edu.cn](mailto:jqwei@tsinghua.edu.cn)


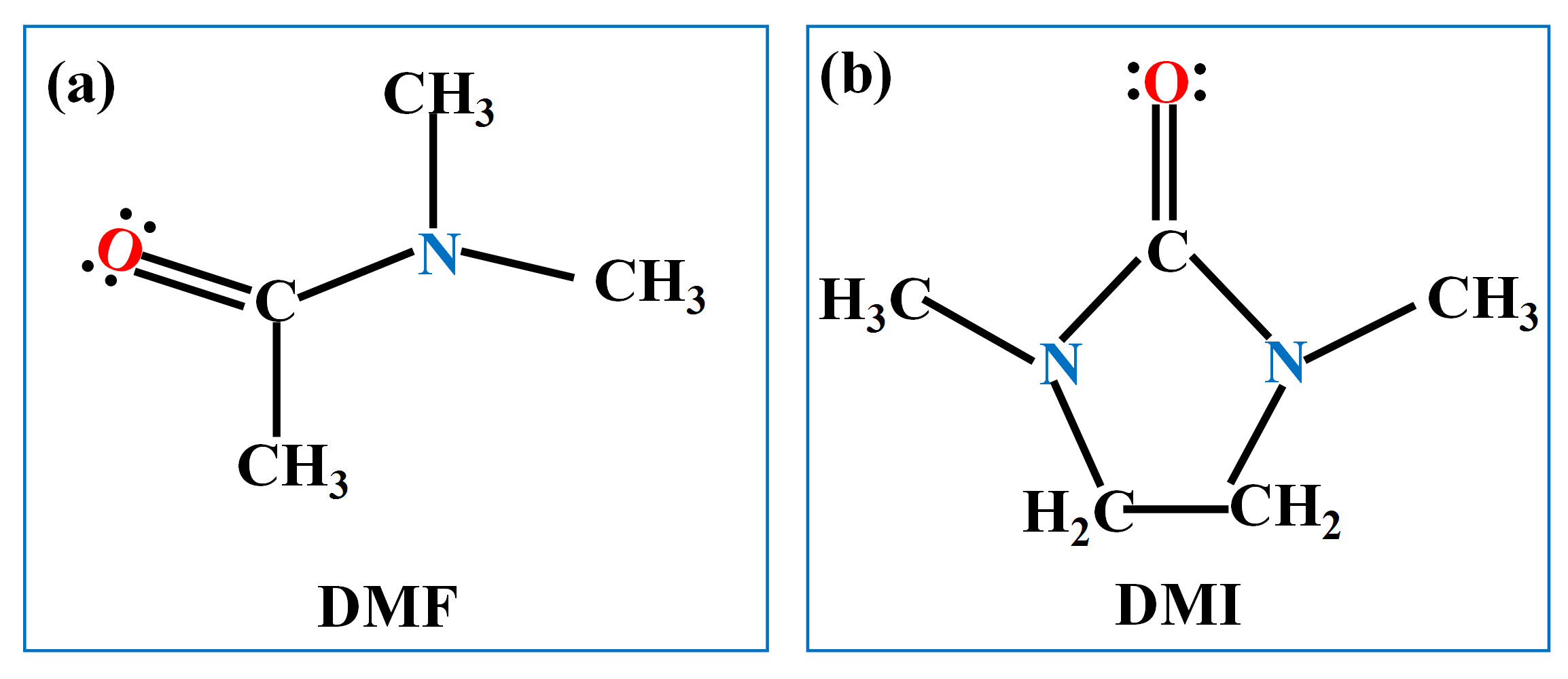


**Figure S1**. Molecular structures of DMF and DMI.


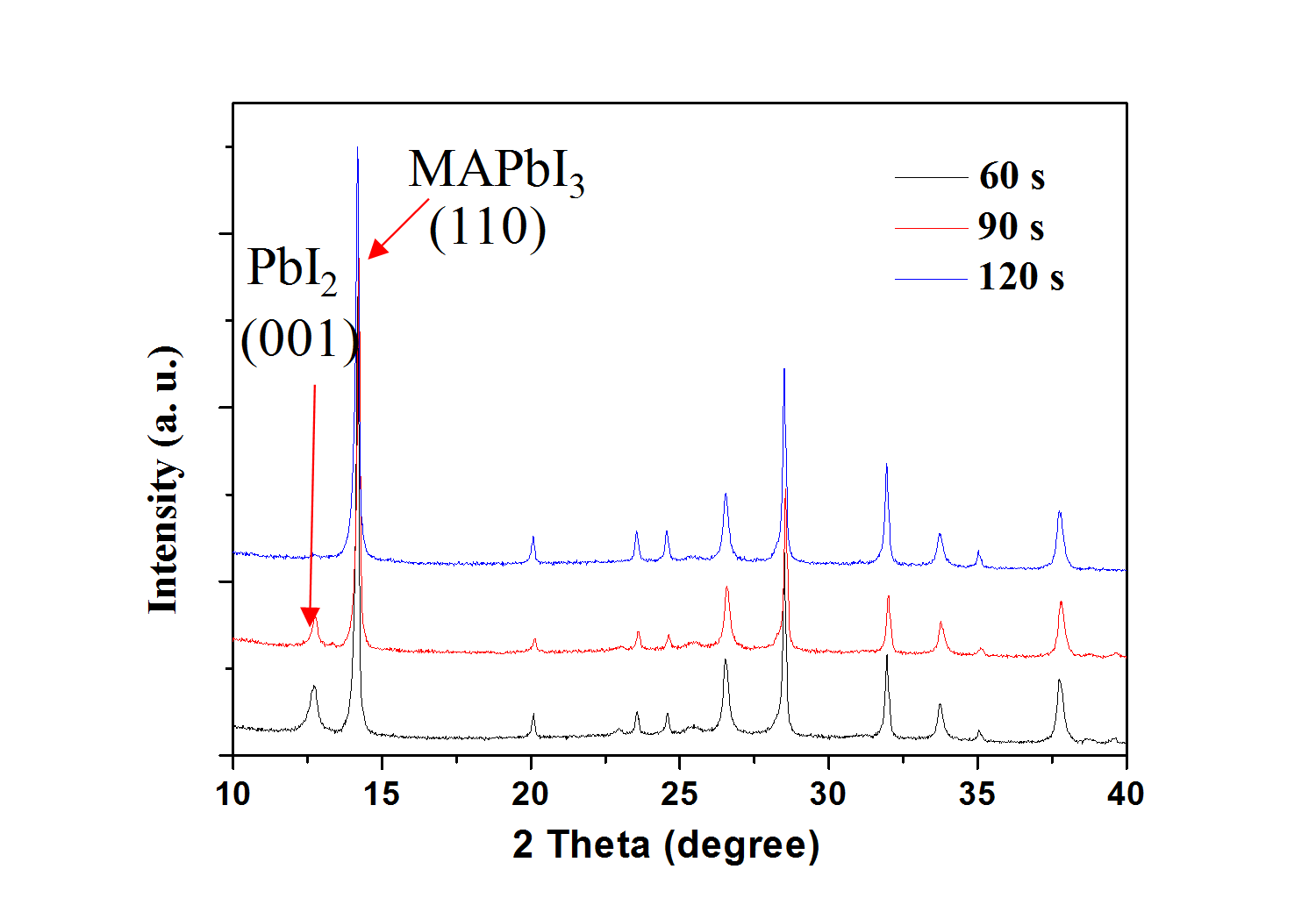


**Figure S2.** XRD curves of the perovskite films from different immersing times.


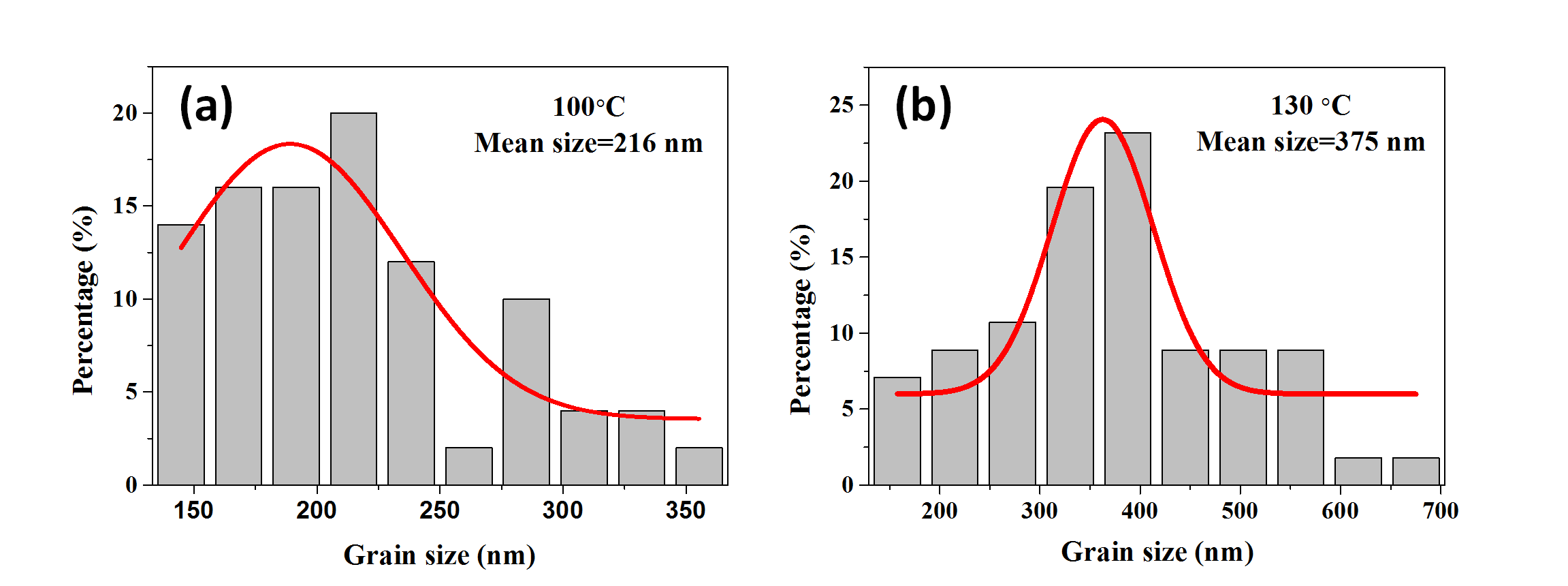


**Figure S3.** Statistical graphs of the perovskite grains prepared by annealing at different temperature. (a) 100 ºC, (b) 130 ºC.


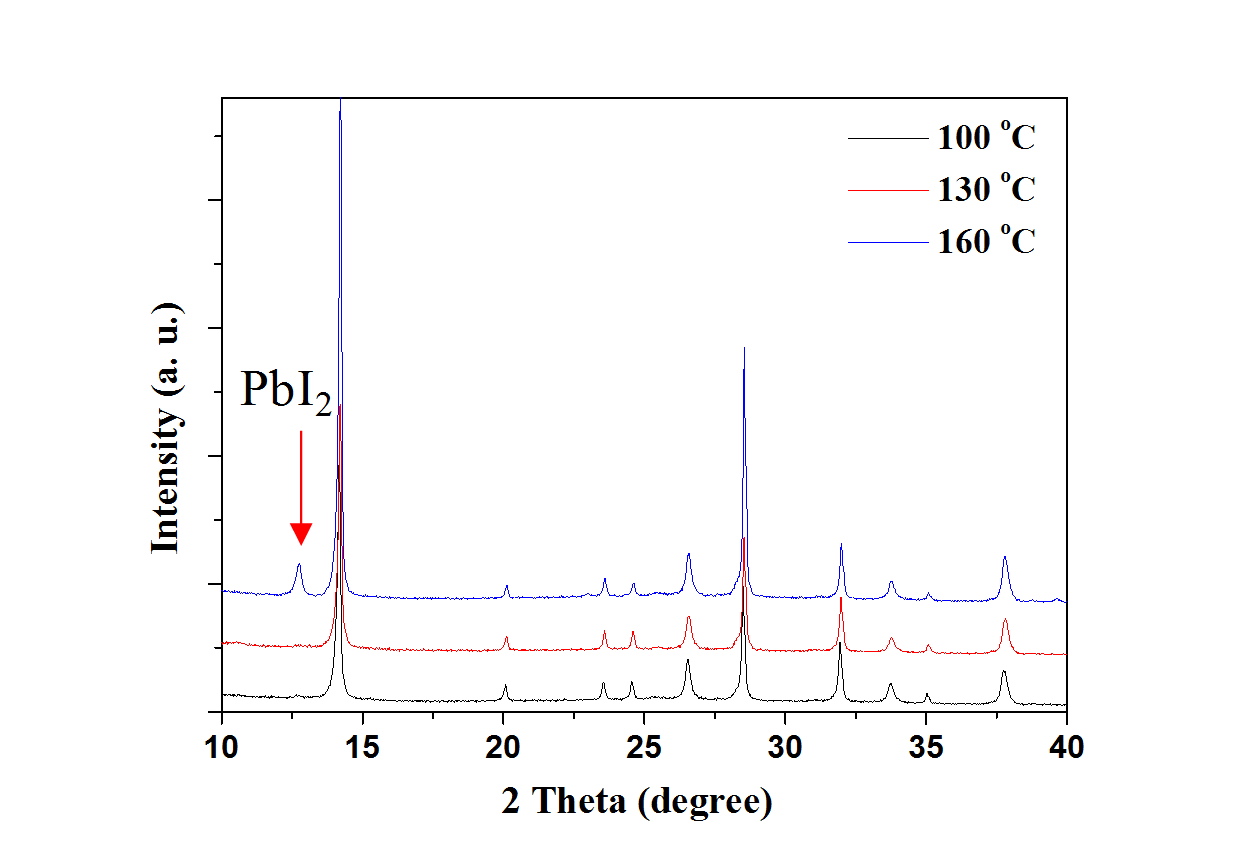


**Figure S4.** XRD curves of the perovskite films prepared from three different annealing temperature.


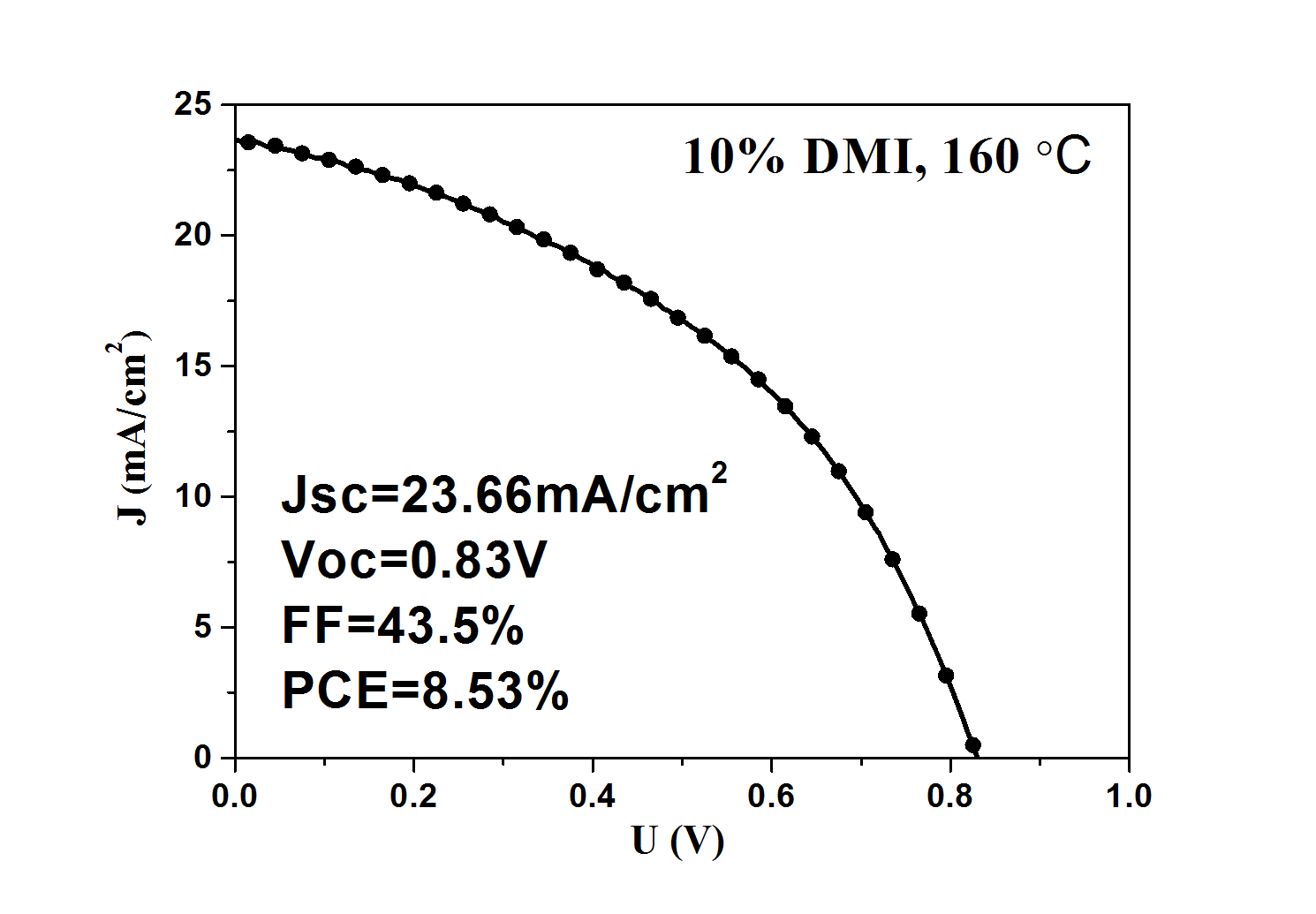


**Figure S5.** J-V curve of the best PSC fabricated from DMI solution and annealing at 160 ºC.


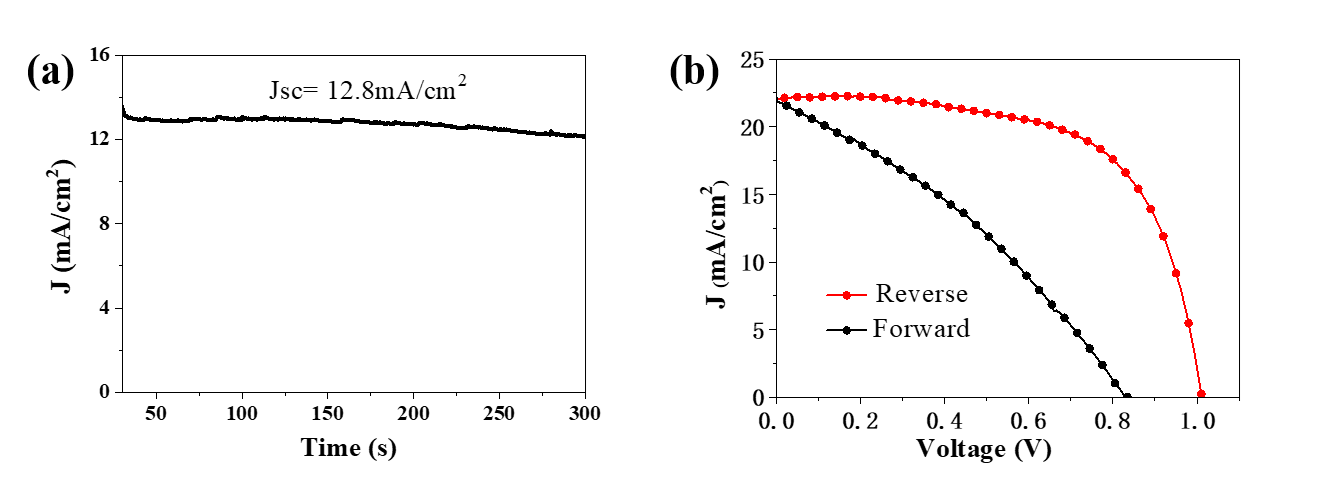


**Figure S6.** (a) Steady-state current measured at the maximum power point (0.78 V), and (b) J-V curves under forward (black line) and reverse (red line) scans for a typical perovskite solar cell.
